# *In vivo* effects of mutant *RHOA* on tumor formation in an orthotopic inoculation model

**DOI:** 10.3892/or.2019.7300

**Published:** 2019-09-03

**Authors:** Takashi Nishizawa, Kiyotaka Nakano, Etsuko Fujii, Daisuke Komura, Yoshie Kuroiwa, Chisako Ishimaru, Makoto Monnai, Hiroyuki Aburatani, Shumpei Ishikawa, Masami Suzuki

**Affiliations:** 1 Department of Research, Forerunner Pharma Research Co., Ltd., Komaba Open Laboratory, The University of Tokyo, Tokyo 153-8904, Japan; 2 Chugai Pharmaceutical Co., Ltd., Kamakura, Kanagawa 247-8530, Japan; 3 Department of Genomic Pathology, Medical Research Institute, Tokyo Medical and Dental University, Tokyo 113-8510, Japan; 4 Chugai Research Institute for Medical Science Co., Ltd., Kamakura, Kanagawa 247-8530, Japan; 5 Genome Science Division, Research Center for Advanced Science and Technology, The University of Tokyo, Tokyo 153-8904, Japan

**Keywords:** RHOA, orthotopic inoculation, diffuse gastric cancer, mutation, gene set enrichment analysis

## Abstract

Ras homolog family member A (*RHOA*) mutations are driver genes in diffuse-type gastric cancers (DGCs), and we previously revealed that *RHOA* mutations contribute to cancer cell survival and cell migration through their dominant negative effect on Rho-associated kinase (ROCK) signaling *in vitro*. However, how *RHOA* mutations contribute to DGC development *in vivo* is poorly understood. In the present study, the contribution of *RHOA* mutations to tumor morphology was investigated using an orthotopic xenograft model using the gastric cancer cell line MKN74, in which wild-type (WT) or mutated (Y42C and Y42S) *RHOA* had been introduced. When we conducted RNA sequencing to distinguish between the genes expressed in human tumor tissues from those in mouse stroma, the expression profiles of the tumors were clearly divided into a Y42C/Y42S group and a mock/WT group. Through gene set enrichment analysis, it was revealed that inflammation- and hypoxia-related pathways were enriched in the mock/WT tumors; however, cell metabolism- and cell cycle-related pathways such as Myc, E2F, oxidative phosphorylation and G2M checkpoint were enriched in the Y42C/Y42S tumors. In addition, the gene set related to ROCK signaling inhibition was enriched in the *RHOA*-mutated group, which indicated that a series of events are related to ROCK inhibition induced by *RHOA* mutations. Histopathological analysis revealed that small tumor nests were more frequent in *RHOA* mutants than in the mock or WT group. In addition, increased blood vessel formation and infiltration of macrophages within the tumor mass were observed in the *RHOA* mutants. Furthermore, unlike mock/WT, the *RHOA*-mutated tumor cells had little antitumor host reaction in the invasive front, which is similar to the pattern of mucosal invasion in clinical *RHOA*-mutated DGC. These transcriptome and pathological analyses revealed that mutated *RHOA* functionally contributes to the acquisition of DGC features, which will accelerate our understanding of the contribution of *RHOA* mutations in DGC biology and the development of further therapeutic strategies.

## Introduction

Diffuse-type gastric cancers (DGCs), which are characterized by poorly differentiated adenocarcinoma that lack cell-cell adhesion and infiltrate into the stroma as single or clustered cells without glandular architecture (1,2), show worse prognosis than the intestinal type (2,3). A characteristic genetic alteration of DGC is the Ras homolog family member A (*RHOA*) missense mutation that is reported in 14–25% of DGC patients (4–6).

A previous report, which evaluated the clinicopathological features of 87 DGC patients by comparing the morphological features of *RHOA*-mutated and wild-type tumors, revealed a distinct permeative intramucosal growth pattern in the mutated tumors (7). RHOA has various biological functions, such as cytokinesis, cell motility and tissue development (8,9). Recently, we revealed that *RHOA* mutations contribute to cancer cell survival and cell migration through their dominant negative effect on the Rho-associated kinase (ROCK) pathway (10), but little is understood of how these functions are related to the clinicopathological features of DGC.

Thus, the present study was designed to evaluate the relationship between the features of DGC and *RHOA* mutations *in vivo*. To this end, we first considered which model was most suited for our evaluation. Orthotopic inoculation is reported to be more likely to reproduce the histopathology of clinical tumors compared to a subcutaneous model (11–13). Our own previous study using a *RHOA*-mutated cancer cell line supported these reports by revealing that, compared with subcutaneous models, orthotopic models exhibited abundant stroma and an invasive character (14). This information prompted us to study the effects of *RHOA* mutations *in vivo* by inoculating the tumor cells into the stomachs of SCID mice.

To understand the molecular mechanism of the effects of *RHOA* mutations, the tumor microenvironment must be analyzed, as both cancer and stromal cells play key roles in forming the tumor microenvironment (15). Therefore, we decided to carry out a transcriptome analysis using next generation sequencing technology, which makes it possible to distinguish human (tumor cells) and mouse (stromal cells) sequences (14,16–18). Thus, in the present study the effects of mutant *RHOA* were evaluated by combining transcriptome analysis of the tumor and stromal components and pathological analysis using an orthotopic xenograft model.

## Materials and methods

### 

#### Cell lines

The human gastric cancer cell line MKN74 (19) was purchased from the cell bank of the Japanese Collection of Research Bioresources (National Institutes of Biomedical Innovation, Health and Nutrition, Osaka, Japan). It was cultured using RPMI-1640 medium (Sigma-Aldrich; Merck KGaA) supplemented with 10% heat-inactivated fetal bovine serum (FBS; Sigma-Aldrich; Merck KGaA), 10 mM HEPES (Gibco; Thermo Fisher Scientific, Inc., Waltham, MA, USA), 1 mM sodium pyruvate (Gibco; Thermo Fisher Scientific, Inc.) and 2.5 g/l D-glucose (Sigma-Aldrich; Merck KGaA). The cells were maintained in a humidified incubator at 37°C with 5% CO_2_.

#### Generation of MKN74 cell lines expressing RHOA mutations

The methods to establish MKN74 cell lines expressing *RHOA* mutations were previously described (10). In brief, the coding sequences for the *RHOA* mutation (NCBI RefSeq Sequence: NM_001664.3) were inserted into the pLVSIN-CMV vector (Takara Bio Inc., Shiga, Japan). The mixture of expression vector and ViraPower Lentiviral Packaging Mix (Thermo Fisher Scientific, Inc.) was introduced into Lenti-X 293T cells (Takara Bio Inc.) using FuGENE HD Transfection Reagent (Promega Corp., Madison, WI, USA). After 48 h, the culture medium was harvested and virus particles were concentrated with Lenti-X Concentrator (Takara Bio Inc.). Prepared lentivirus was transfected into each cell line with hexadimethrine bromide (final 8 µg/ml; Sigma-Aldrich; Merck KGaA). Hygromycin (Thermo Fisher Scientific, Inc.) was added to establish stable transfectants at a final concentration of 25 µg/ml for MKN74. The *RHOA* cDNA introduced to the MKN74 cells have mutations that cause resistance to *RHOA*-siRNA. Thus, we confirmed the expression of exogenous RHOA by western blot analysis after *RHOA*-siRNA treatment to eliminate endogenous RHOA which was hindering detection of the transgenes (10). These cells showed comparable cell growth *in vitro* (Fig. S1). As for other *in vitro* profiles, we reported the cell motility and actin stress fiber formation in our previous study (10) and the features are summarized in Table I.

#### Cell growth assays

Cells (1.0×10^3^/100 µl/well) were seeded in 96-well cell culture plates (TPP; Sigma-Aldrich; Merck KGaA) in triplicate. The viable cells were measured 1 day, 4 and 7 days after cell seeding using the CellTiter-Glo 3D Cell Viability Assay, according to the manufacturer's protocol (Promega Corp.). The luminescence was measured using a plate reader (PerkinElmer, Inc., Waltham, MA, USA).

#### Animals

Seven-week-old male severe combined immune- deficient (SCID) mice (C.B-17/lcr-*scid*/*scid* Jcl) were provided by CLEA Japan, Inc. (Tokyo, Japan). All animals were housed in a specific pathogen-free environment under controlled conditions (temperature, 20–26°C; humidity, 30–70%; light/dark cycle, 12/12 h) and were allowed to acclimatize and recover from shipping-related stress for more than 5 days prior to the study. Chlorinated water and irradiated food were provided *ad libitum*. The health of the mice was monitored by daily observation. The humane endpoints were deterioration of general conditions and sacrifice in the event of a body weight loss exceeding 20%. All animal experiments were performed at Chugai Pharmaceutical Co., Ltd. The experiments were reviewed and approved by the Chugai Pharmaceutical Co., Ltd., Institutional Animal Care and Use Committee.

#### Orthotopic inoculation and tissue sampling

**Figure 1.**
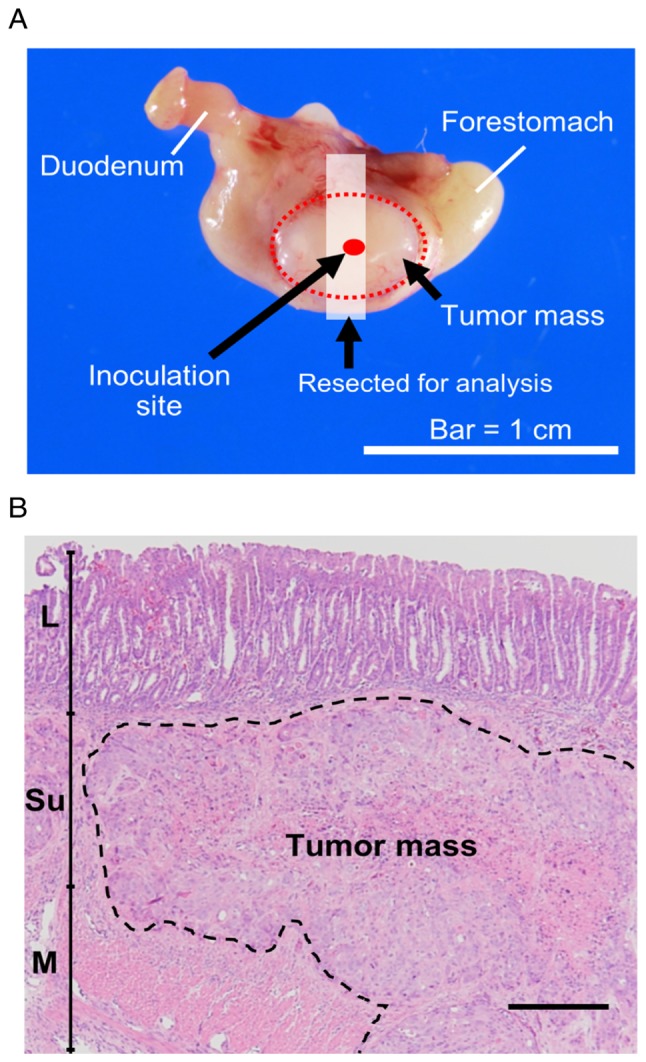
(A) Illustration of the injection site. (B) Representative figure showing the location of engraftment. L, lamina propria mucosae; Su, submucosa; M, muscular layer. Scale bar, 250 µm.

The mice were inoculated with 3×10^4^ cells, suspended in 20 µl of RPMI-1640 medium containing 50% Matrigel (Corning Inc., Corning, NY, USA). Transplantation was carried out using a method based on previous studies (14,20,21). Briefly, the animals were anesthetized under 2.5% isoflurane inhalation anesthesia. Then a surgical incision was made in the medial abdomen and the stomach was exposed. Next 20 µl of cells suspended in 50% Matrigel were inoculated into the serosa of the ventral stomach (Fig. 1A). Finally, the stomach was returned to the original position, and the incision was closed. Inoculation was defined as successful when cells had been injected into the intended area with no major leakage outside of the stomach wall. For the wild-type (WT) and mutant groups, the procedure was performed until there were 5 mice for each group. With the mock group, 8 mice were included as controls. The total number of mice used in the study was 42 (Mock, 8; WT, 10; Y42C, 12; Y42S, 12), and the success rate for the inoculation procedure was 55% (23/42 mice: Mock, 8/8; WT, 5/10, Y42C, 5/12; Y42S, 5/12). The average body weight of each group was Mock, 25.9±0.5 g; WT, 25.0±1.1 g; Y42C, 26.2±1.0 g; Y42S, 24.8±1.4 g. The largest diameter of the tumors measured from the serosal side of the stomach was 1.0 cm. There was no difference in diameter between groups. The tumors were observed as single nodules with no multiple tumors. The tumors were sampled at 4 weeks after inoculation. At necropsy the animals were sacrificed under isoflurane inhalation anesthesia by exsanguination from the abdominal artery and grossly examined. Histopathologically, the tumors were engrafted as an extension from the submucosa to the muscular layer (Fig. 1B). The tumors subjected to histopathology and transcriptome sequencing are listed in Table SI.

#### RNA preparation and transcriptome sequencing

The tumor tissues were collected in Biomasher III (Fujifilm Wako Pure Chemical Corp., Osaka, Japan). We added TRIzol reagent (Thermo Fisher Scientific, Inc.) into the tube and mashed the tissues with a pestle. The tissue lysate was obtained after centrifugation (12,000 × g for 2 min). Total RNA was extracted using the RNeasy mini kit (Qiagen, Hilden, Germany). Total RNA (1.7–2.0 µg) was used to prepare a transcriptome sequencing library for each tumor sample using TruSeq stranded mRNA Library Prep kit (Illumina, San Diego, CA, USA) following the manufacturer's directions. The libraries were sequenced in 100 bp paired-end reads on a HiSeq2500 sequencer (Illumina). Six libraries were loaded into the single lane of an Illumina flow cell, producing more than 50 million paired-end reads for each sample. Sequenced reads were mapped to all RefSeq transcripts of human (hg38 coordinates) and mouse (mm 10 coordinates) using bowtie 1.1.2 (22) allowing up to one mismatch, and reads mapped to both species or to multiple genes were discarded. The remaining reads were used to estimate the gene expression profile of human cancer cells and mouse stroma cells according to the methods as previously described (18). Gene expression values were normalized for cancer cells and stromal cells independently so that the sum of the expression values below the 95th percentile would be 300,000. Samples with human (cancer) reads <5% or >95% were removed for subsequent analysis (Table S1).

#### Unsupervised clustering of gene expression profiles

After gene-wise Z-score transformation, hierarchical clustering was performed using a Euclidean distance metric on the expression of the highly and variably expressed genes across all samples (mean normalized expression >3.0 and coefficient of variation >30%) using the ComplexHeatmap Bioconductor package (23).

#### Differential expression analysis

The DESeq2 R package (24) was used for cancer cells and stromal cells independently to detect genes that were expressed differentially between the two conditions. Raw count detected by CASTIN algorithm was used as the input for the DESeq2 software. Adjusted P-values were used to detect differentially expressed genes and log2 fold change shrinkage was used to rank genes for Gene Set Enrichment Analysis (GSEA).

#### GSEA analysis

GSEA (25,26) was used to identify gene sets that were altered between two conditions. After sorting the genes based on the log2 fold change, we applied a pre-ranked GSEA with the javaGSEA desktop application (http://software.broadinstitute.org/gsea/downloads.jsp). As gene sets, we used hallmark gene sets in The Molecular Signature Database or genes significantly upregulated or downregulated in the presence of a ROCK inhibitor (Y-27632) in human keratinocytes (Table SIIA and B) (27).

#### Pathological sample preparation

The tumor tissues were sampled and fixed in 4% paraformaldehyde at 4°C for 24 h and embedded into paraffin using the AMeX method (28,29). Thin sections were prepared at a thickness of 3–4 µm, and hematoxylin and eosin stains and Sirius red stains were performed by routine methods for histopathological evaluation. Additional slides were used for immunohistochemistry for CD31 and F4/80. The primary antibodies were rabbit polyclonal antibody to CD31 (1:100 dilution; cat. no. ab28364; Abcam, Cambridge, UK), and rat monoclonal antibody to mouse macrophage F4/80 antigen (1:100 dilution; clone BM8; BMA Biomedicals, Augst, Switzerland). Briefly, after deparaffinization, the slides were treated for antigen retrieval by autoclave heating at 120°C for 10 min for CD31, and proteinase K (Dako; Agilent Technologies) for F4/80. Then endogenous peroxidase was quenched with 1% H_2_O_2_ in methanol, followed by blocking with skim milk (Thermo Fisher Scientific, Inc.). The primary antibodies were incubated with the slides at 4°C overnight. Finally, the secondary antibodies (LSAB2, Agilent Technologies, or N-Histofine^®^ simple stain mouse Rat MAX-PO; Nichirei Biosciences Inc., Tokyo, Japan) were applied and the reactions were visualized by 3,3′-diaminobenzidine. The slides were counterstained with hematoxylin and coverslipped for reading under a light microscope.

#### Histological evaluation

The slides were read and reviewed by 2 certified pathologists, and the scoring criteria were determined by discussion between the pathologists. Furthermore, scoring was carried out based on this criteria by the following methods. The ratio of the area of small nests to total tumor area was evaluated by image analysis on virtual slides scanned using the Leica Aperio ScanScope AT2 (Leica Biosystems, Wetzlar, Germany) and analyzed with the Aperio Image Scope software (version 12.3.2.7001; Leica Biosystems). To score CD31, the density of positive vascular structures per site at ×20 magnification was evaluated by the following criteria: 0, not observed; 1, >0–6 per site; 2, >6–9 per site; 3, 10 or more. For F4/80, the density of positive cells within the tumor mass was evaluated according to the following criteria: 0, not observed; 1, scattered; 2, diffuse; 3, focally dense. Additionally, the histopathological findings in the invasive front of the tumor mass were scored. Each main finding (fibrosis, inflammatory cell infiltration, necrosis of tumor cells) was graded according to the following criteria: 0, not observed; 1, occasionally observed; 2, moderately observed; 3, frequently observed. Then the sum of the 3 findings for each animal was calculated and designated as the histology score.

#### Statistical analysis

The statistical analysis was performed using the JMP statistical software program (version 11; SAS Institute, Cary, NC, USA). Analysis for the ratio of small nest area was conducted by one-way analysis of variance (ANOVA) followed by a Dunnett's multiple comparison post hoc test. The comparisons of the histologic scores were assessed with non-parametric Steel's test. P<0.05 was considered to indicate a statistically significant difference.

## Results

### 

#### RHOA mutants were found to be enriched in distinctly differential pathways when compared to mock/WT and showed inhibition of ROCK signaling in vivo

**Figure 2.**
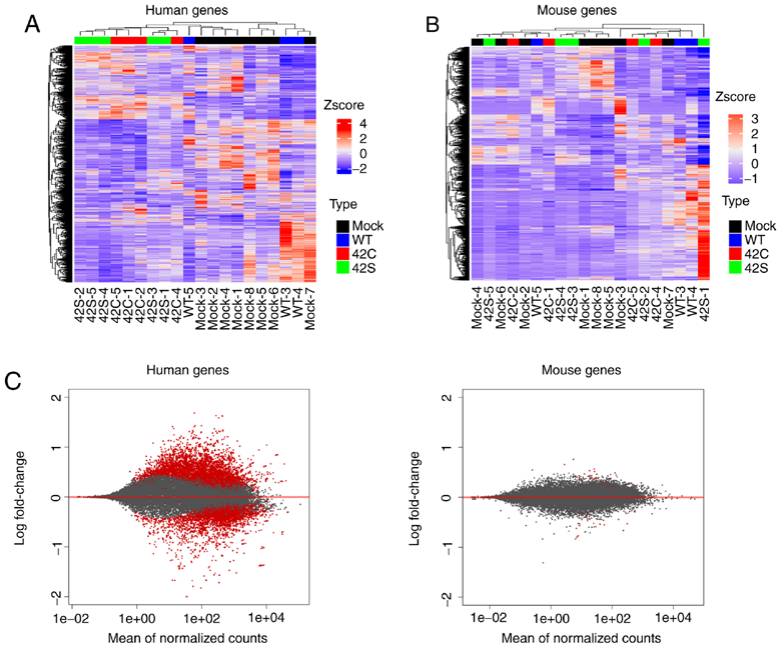
Transcriptome analysis of orthotopic inoculated tumors. Hierarchical clustering of the differentially expressed human genes (A) and mouse genes (B) across all samples is shown vertically for genes and horizontally for the tumor samples. Samples that are mock, wild-type (WT), Y42C, and Y42S are indicated in black, blue, red, and green, respectively. In the matrix table, red indicates high expression and blue indicates low expression profiles. (C) MA plots of altered gene expression between the Y42C/Y42S group and mock/WT group in human genes (tumor, left panel) and mouse genes (stroma, right panel). Each dot represents a transcript. The x-axis shows normalized counts and the y-axis shows the expressional change in log scale. Transcripts with an adjusted P-value <0.1 are shown in red.

To determine the effects of *RHOA* mutations *in vivo*, we introduced WT, Y42C, and Y42S *RHOA* into the MKN74 gastric cancer cell line, which originally has WT-*RHOA*. To evaluate the expression profile of cells that had been inoculated into the mouse stomach, the RNA of each tumor tissue was eluted and sequenced to obtain tumor (human) and host (mouse) transcriptome data simultaneously. Unsupervised hierarchical clustering for human expression data showed that it was clearly divided into two groups: The Y42C/Y42S group and the mock/WT group (Fig. 2A). We also performed unsupervised hierarchical clustering for mouse expression data, but as the groups were allocated to various clusters, differences in expression profiles between groups could not be identified (Fig. 2B). Additionally, the level of expressional change in stroma (mouse) was much lower than that in the tumor (human) (Fig. 2C). Next we compared the expression of endothelial (*Cd31*), macrophage (*Adgre1, Cd68, Itgax, Mrc1*), and fibroblast (*Col1A1, Thy1, Acta2, S100a4*) markers, but there was no difference between the groups (data not shown). From this expression profile we judged that further analysis should be focused on the expression profile of tumor cells.

**Figure 3.**
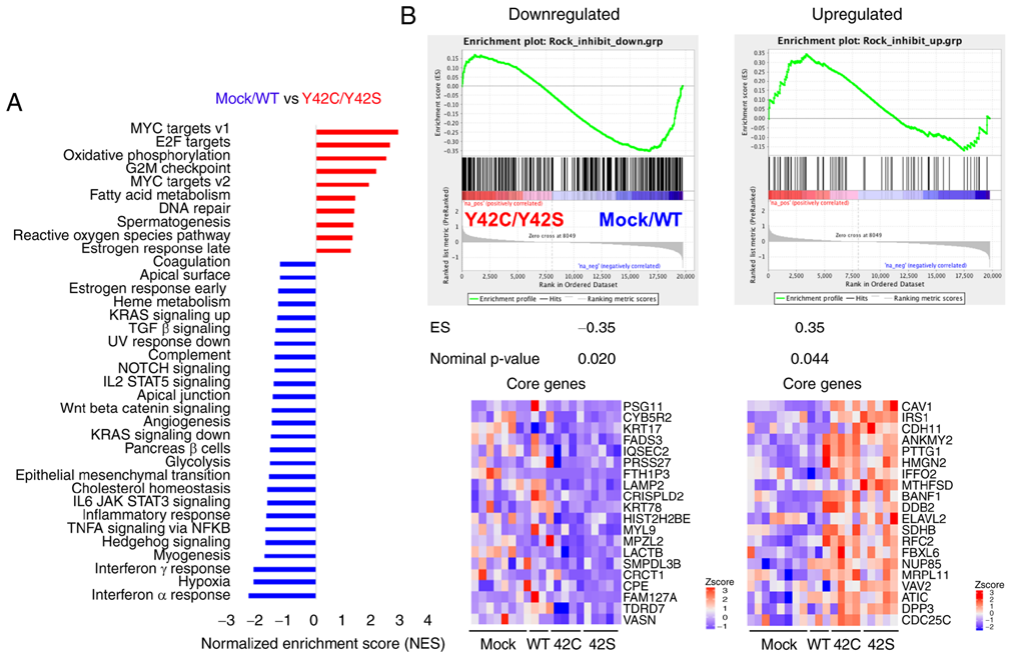
GSEA analysis of mock/WT vs. Y42C/Y42S. (A) GSEA analysis using hallmark gene sets from the Molecular Signature Database (see: http://software.broadinstitute.org/gsea/msigdb/index.jsp) was carried out. The statistically significant signatures were selected (FDR <0.25) and placed in order of normalized enrichment score (NES), which represents the strength of the relationship between the phenotype and gene signature. Red bars indicate the pathways enriched in the Y42C/Y42S group and blue bars indicate those enriched in the mock/WT group. (B) GSEA results of the correlation between gene sets in the two groups and the gene signatures reported after treatment with a ROCK inhibitor. The GSEA results for downregulated genes are in the left panel, and for upregulated genes in the right panel. In each enrichment plot, the green curve corresponds to the enrichment score (ES) curve, which is the running sum of the weighted ES. The nominal P-value estimates the statistical significance of a single gene set's enrichment score. Heat maps show the top 20 core genes (ranked by ‘Rank Metric Score’, which is the signal to noise ratio for each gene used to position the gene in the ranked list) that drive the enrichment score of the GSEA clusters. Heat maps of the total core genes are shown in Fig. S3. GSEA, Gene Set Enrichment Analysis; ROCK, Rho-associated kinase.

To understand the state of tumor cells in mutant and non-mutant groups, we performed GSEA (25,26). Pathways related to hypoxia and inflammation such as interferon α/γ, TNFα, IL6_JAK_STAT3, and to inflammatory response were enriched in the mock/WT group (Figs. 3A and S2A). On the other hand, Myc, E2F, oxidative phosphorylation, and G2M checkpoint pathways, which are related to cell cycle or cell metabolism, were enriched in the Y42C/Y42S group (Figs. 3A and S2B). In addition, we confirmed the ROCK signaling status in the tumor cells. To evaluate the activation status of ROCK signaling, we performed a GSEA analysis with a ROCK inhibitor-related gene set, which was selected from published data (Table SIIA and B) (27). As a result, genes downregulated after ROCK inhibitor treatment were significantly enriched in the mock/WT group, whereas the upregulated genes were enriched in the Y42C/Y42S group (Figs. 3B and S3). These results indicated that ROCK signaling was inhibited in *RHOA* mutants *in vivo* as well as *in vitro*.

#### Mutated RHOA contributes to a pattern of small tumor nest growth, and to changes in stromal cells

**Figure 4.**
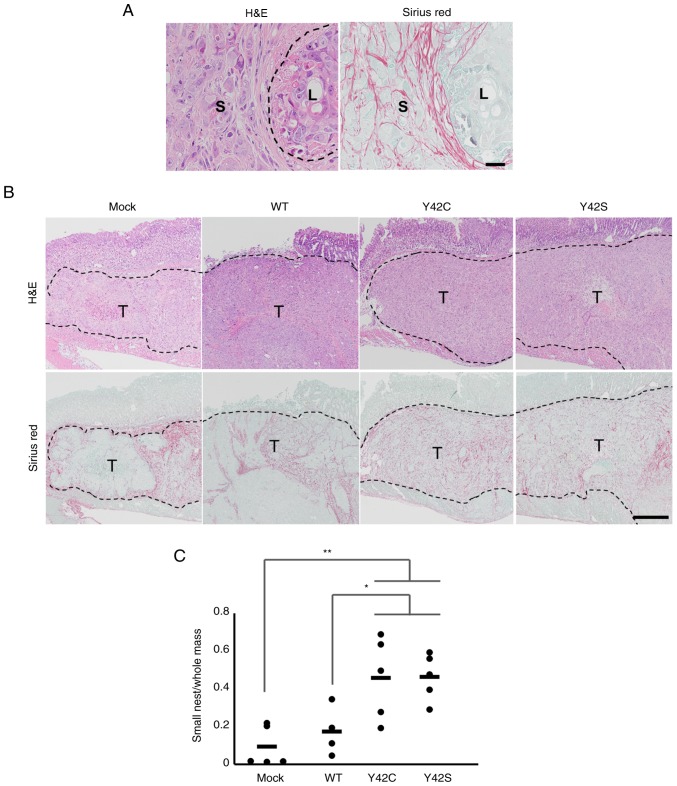
Histopathological evaluation of the formation of small tumor nests. (A) Representative images of small (S) and large (L) tumor nest areas. H&E, hematoxylin and eosin stain (left), and Sirius red stain (right). Scale bar, 100 µm. (B) Representative images of the tissue sections in mock, WT and *RHOA* mutants. The tumor nests are circumscribed by collagen fibers. T, tumor area. Scale bar, 1 mm. H&E, hematoxylin and eosin stain (upper row), and Sirius red stain (bottom row). (C) The ratio of small tumor nest area to total tumor area. Each dot represents the ratio in a tumor tissue section from 1 animal. The bars show the average for each group. *P<0.05, **P<0.01, one-way analysis of variance followed by a Dunnett's test. WT, wild-type; *RHOA*, Ras homolog family member A.

In the orthotopic model, the size of the tumor cannot be compared accurately because the size is affected by the area of inoculation. Because of this we compared the expression of Ki-67 but found that there was no difference between the WT group and mutant groups. Thus, we conducted a detailed histopathological analysis and compared the morphologic features of the tumor with the RNA expression profiles. Morphologically, mock and WT tumors consisted mainly of large tumor nests, but in contrast, the mutant tumors consisted mainly of small tumor nests that were circumscribed by fine collagen fibers (Fig. 4A and B). This was further confirmed by morphometric analysis of the area for each type of tumor nest. The ratio of small tumor nest to total tumor nest area in Y42C and Y42S was significantly higher than in mock or WT (Fig. 4C). The average ratio of small tumor nests was 0.09 in mock, 0.17 in WT, 0.46 in Y42C, and 0.46 in Y42S. Thus we found that the mutant tumors had a distinctly different growth pattern compared to the mock or WT tumors.

We speculated that the difference in the amount of small tumor nests was related to a difference in tumor-stromal interaction, and because the hypoxia signature was enriched in mock/WT but not in *RHOA* mutants, we focused on tumor angiogenesis.

**Figure 5.**
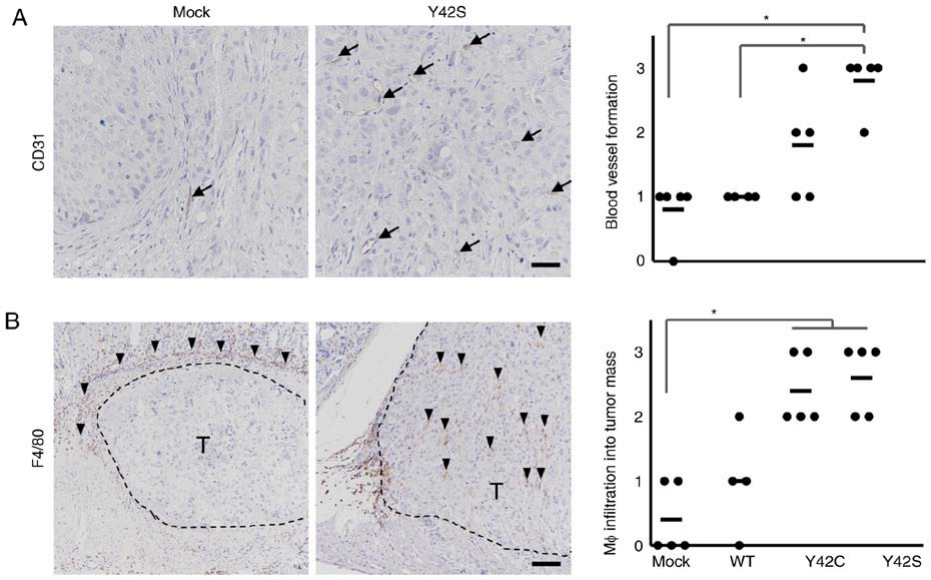
Immunohistochemical analysis of stromal components. Representative images of immunohistochemical staining for endothelial cells (A, CD31, arrows; scale bar, 100 µm) and macrophages (Mφ) (B, F4/80, arrowheads; scale bar, 200 µm) are shown. T, tumor area. Scoring criteria for CD31 (×20 magnification): 0, not observed; 1, >0–6 per site; 2, >6–9 per site; 3, 10 or more per site. Scoring criteria for F4/80: 0, not observed; 1, scattered; 2, diffuse; 3, focally dense. In the corresponding histology scores, each dot stands for the score in a tumor tissue section from 1 animal. The bars show the average for each group. *P<0.05, difference between mutant group and control group was assessed with nonparametric Steel's test. WT, wild-type.

In order to determine the involvement of angiogenesis, we evaluated the number of CD31-positive blood vessels by immunohistochemical analysis (Fig. 5A) and found that there were higher numbers in Y42C and Y42S than that in the mock and WT. The average scores for the number of blood vessels per site were 1.8 in Y42C, 2.6 in Y42S, 0.8 in mock, and 1.0 in WT. Tumor angiogenesis is reported to be induced by tumor associated macrophages (30,31), thus next we evaluated macrophage (Mφ) infiltration into tumors by immunohistochemical analysis of F4/80 (Fig. 5B). We found that in the mock and WT tumors, the positive cells tended to be located around the tumor mass, but in mutant tumors, the macrophages tended to diffusely infiltrate the tumor mass. This was further confirmed by scoring of the positive cells infiltrating into the tumor mass. The average scores of macrophage infiltration into the tumor mass were 2.4 in Y42C and 2.6 in Y42S, which were higher than those in the mock (0.4) and WT (1.0) tumors. These results indicated that *RHOA* mutations contributed to tumor angiogenesis and the infiltration of macrophages.

#### Reduced host reaction in the invasive front of RHOA mutant tumors

**Figure 6.**
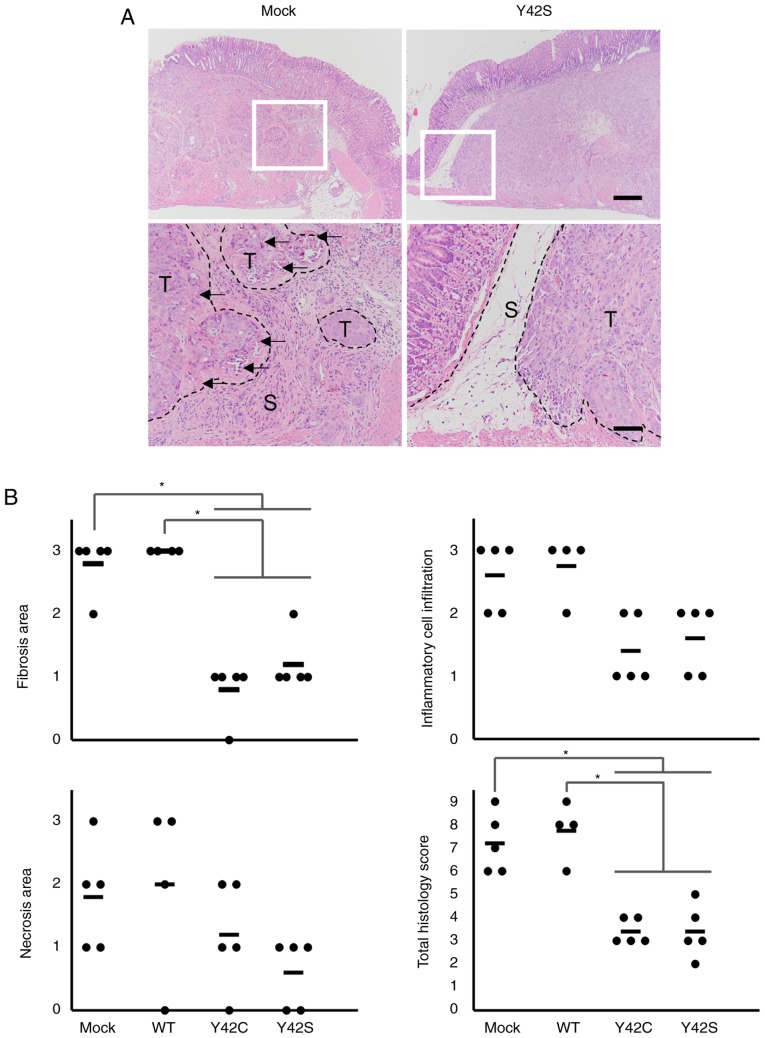
Histological evaluation of the invasive front of tumors using hematoxylin and eosin stain. (A) Representative images of the invasive front of the tumor mass. The areas marked in squares in the upper row are shown at higher magnification in the lower row. The inflammatory cell infiltration with fibrosis observed in the stroma of the mock tumor is markedly weaker in the mutant tumor. S, stroma; T, tumor mass. Arrows show necrosis of tumor cells. Scale bar, 500 µm (upper panels) and 200 µm (lower panels). (B) Scoring for host reaction in the invasive front. Scoring criteria: 0, not observed; 1, occasionally observed; 2, moderately observed; 3, frequently observed. The total histology score is the sum of scores for the other three findings. Each dot stands for the score in a tumor tissue section from 1 animal. The bars show the average for each group. *P<0.05, difference between mutant group and control group was assessed with Steel's test. WT, wild-type.

Next we focused on the invasive front of the tumor mass (Fig. 6A). In the invasive front of the mock and WT tumors, there was a desmoplastic reaction or fibrosis accompanied by inflammatory cell infiltration. Along with these findings, necrosis of tumor cells was increased. In contrast, the host reaction was notably weaker in the mutant tumors. To further clarify the difference in host reaction, the findings were scored and statistically analyzed. We found that the total histology scores in the Y42C and Y42S tumors were significantly lower than scores in the mock/WT tumors (Fig. 6B). The average total histology score of each group was 3.4 in Y42C, 3.4 in Y42S, 7.2 in mock, and 7.8 in WT. These results indicate that *RHOA*-mutant cancer cells have the ability to invade the surrounding tissue without causing a strong antitumor reaction.

## Discussion

In the present study, we revealed the transcriptome and histological changes that occurred when *RHOA* mutations were introduced into MKN74 cells. Tumors in the *RHOA* mutant groups were composed mainly of small tumor nests compared to those in the non-mutant groups. A distinct feature of clinical DGC is that tumor cells exist within the stroma as single cells or small cell clusters. Our current results suggest that *RHOA* mutations at least in part contribute to this poorly cohesive growth pattern, although as non-mutated clinical DGC also exhibits this feature, there may be other factors involved.

Another notable morphological finding in the present study was that, in contrast to mock and WT tumors, *RHOA*-mutated tumors had little host reaction in the invasive front of the tumor. We previously reported that in clinical DGC, *RHOA*-mutated tumors showed an intramucosal permeative growth pattern in the mucosa, which is characterized by infiltration of tumor nests between the normal pits or glands with no recognizable margin, indicating that there is little stromal reaction against the tumor. This contrasted with the expansive pattern of destructive invasion and a relatively well-defined margin seen in non-mutated tumors (7). The lack of host reaction in the *RHOA*-mutant tumors of the present study was thought to reflect the distinctive growth pattern found in the mucosa of clinical *RHOA*-mutated DGC. Together with the effects on the size of the tumor nests, our results suggest that *RHOA* mutations are likely to have a direct role in the development of the morphology that is distinctive of clinical DGC.

Since the hypoxic signature in mock/WT tumors was more enriched than that noted in the mutant tumors, we considered the involvement of angiogenesis and found that the *RHOA* mutants had higher levels of blood vessel formation and infiltration of macrophages into the tumor mass. Angiogenesis is closely related to infiltration of macrophages (32–34). Additionally, Yin *et al* reported that a high density of macrophages was correlated with DGC (35). Therefore, these results suggest that *RHOA* mutants affect tumor angiogenesis induced by macrophages in the tumor mass, and that the tumor microenvironment may be closely related to the growth pattern of DGC.

In our previous *in vitro* study, we found that mutant *RHOA* inhibited ROCK signaling in a dominant negative manner, which caused the actin cytoskeleton to become loose and led to a change in cell-cell interactions (10). Such changes may be related to the growth pattern of small nests *in vivo*. ROCK inhibition is also known to be related to anoikis resistance (36), which may have a role in the maintenance of the small nest pattern. The lack of strong host reaction in the *RHOA*-mutated tumors may also be related to these mechanisms; however, since much is still unknown, further studies are necessary to elucidate the molecular mechanism of the features *in vivo*.

The dramatic difference in host reaction between *RHOA* mutants and mock/WT suggests that *RHOA* mutations affect cells such as fibroblasts, endothelial cells, and immune cells in mouse stroma. However, the level of expressional change in the stroma was much lower than that in the tumor (Fig. 2C), and the mouse expression profiles did not reveal any difference between *RHOA* mutants and mock/WT tumors. This discrepancy between the histopathology results and the RNA expression profile may have occurred because we evaluated the expression in the whole tumor tissue. As there are several cell components in the tissue surrounding the tumor mass, local changes such as those at the invasion front were thought to be difficult to discriminate. To overcome this issue, we are trying to profile the expression at the single cell level instead of in bulk. Since several reports show detailed cross-talk between tumor and components of the tumor microenvironment (37–39), we anticipate that single cell RNA sequencing will more precisely reveal the interaction between the tumor and its microenvironment and the molecular mechanisms involved.

In summary, our results from an orthotopic model in the stomach have provided the first direct evidence concerning the effects of mutated *RHOA in vivo*. Since the features of this xenograft model allow insights into the biology in human clinical cancer, these results will accelerate the understanding of how *RHOA* mutations contribute to the disease biology of DGC and may promote the development of future therapeutic strategies.

## Supplementary Material

Supporting Data

## Figures and Tables

**Table I.** *In vitro* phenotypes of the MKN74 cells used for engraftment.

Transfected *RHOA*	Cell growth rate	Migration activity^a^	Invasion activity^a^	Actin fiber formation^b^
WT	n.s.	Low	n.s.	High
Y42C	n.s.	High	n.s.	Low
Y42S	n.s.	High	n.s.	Low

a Migration and invasion activity were evaluated with Boyden Chamber assay.

b Actin stress fiber formation was evaluated with rhodamine phalloidin staining as previously described (10). n.s., no significant difference; WT, wild-type; *RHOA*, Ras homolog family member A.

## Data Availability

The datasets used during the present study are available from the corresponding author on reasonable request.
