# Supplementary material for: In vivo effects of mutant RHOA on tumor formation in an orthotopic inoculation model
Source: Oncol Rep. 2019 Sep 3;42(5):1745–54. doi: 10.3892/or.2019.7300 (PMC6775816; doi:10.3892/or.2019.7300)
Supplement: Supporting Data [file Supplementary_Data.pdf]

Figure S1. Cell growth rate of WT-, Y42C-, and Y42S-transfected MKN74 cells compared to the growth rate of the mock group. Cell lines were seeded in a cell culture plate and then incubated for 7 days. The viable cells were measured with a CellTiter-Glo 3D Cell Viability Assay on day 1, 4 and 7. Data are shown as mean ± SD (n=3). WT, wild-type.

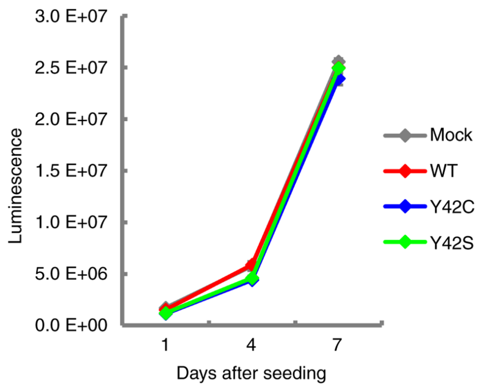

Figure S2. Gene Set Enrichment Analysis (GSEA) of the mock/WT cohort vs. Y42C/Y42S cohort using hallmark gene sets from the Molecular Signature Database. The enrichment plots have been categorized into those statistically significant signatures in the mock/WT group (A), and those in the Y42C/Y42S group (B). Each enrichment plot illustrates the specific gene sets associated with the difference between two cohorts.

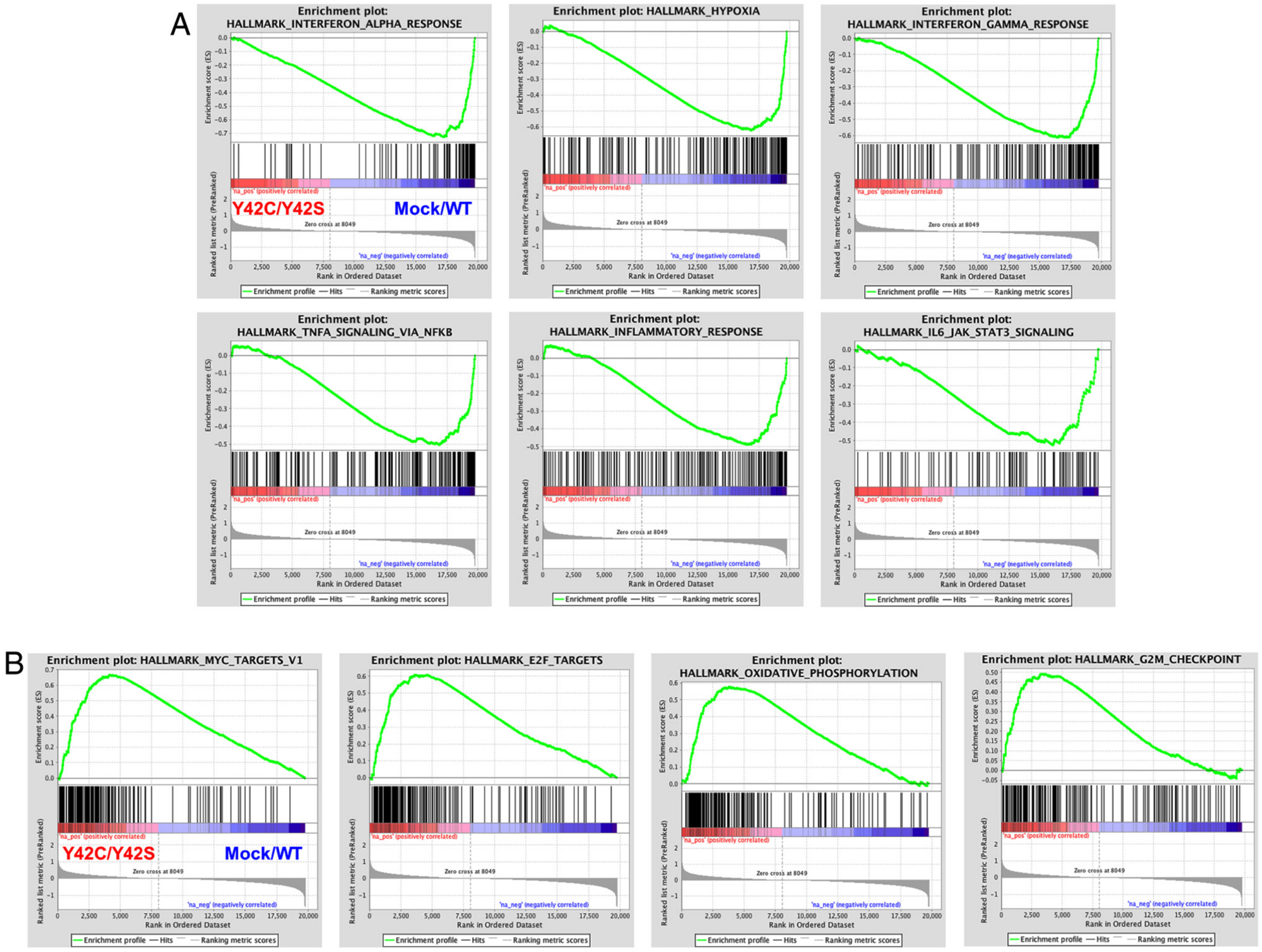

Figure S3. Heat maps of a Gene Set Enrichment Analysis (GSEA) of mock/WT vs. Y42C/Y42S using the gene signatures reported after treatment with a ROCK inhibitor. The genes include all the core genes that drive the enrichment score of the GSEA clusters. The results for downregulated genes are in the left panel, and for upregulated genes in the right panel. WT, wild-type.

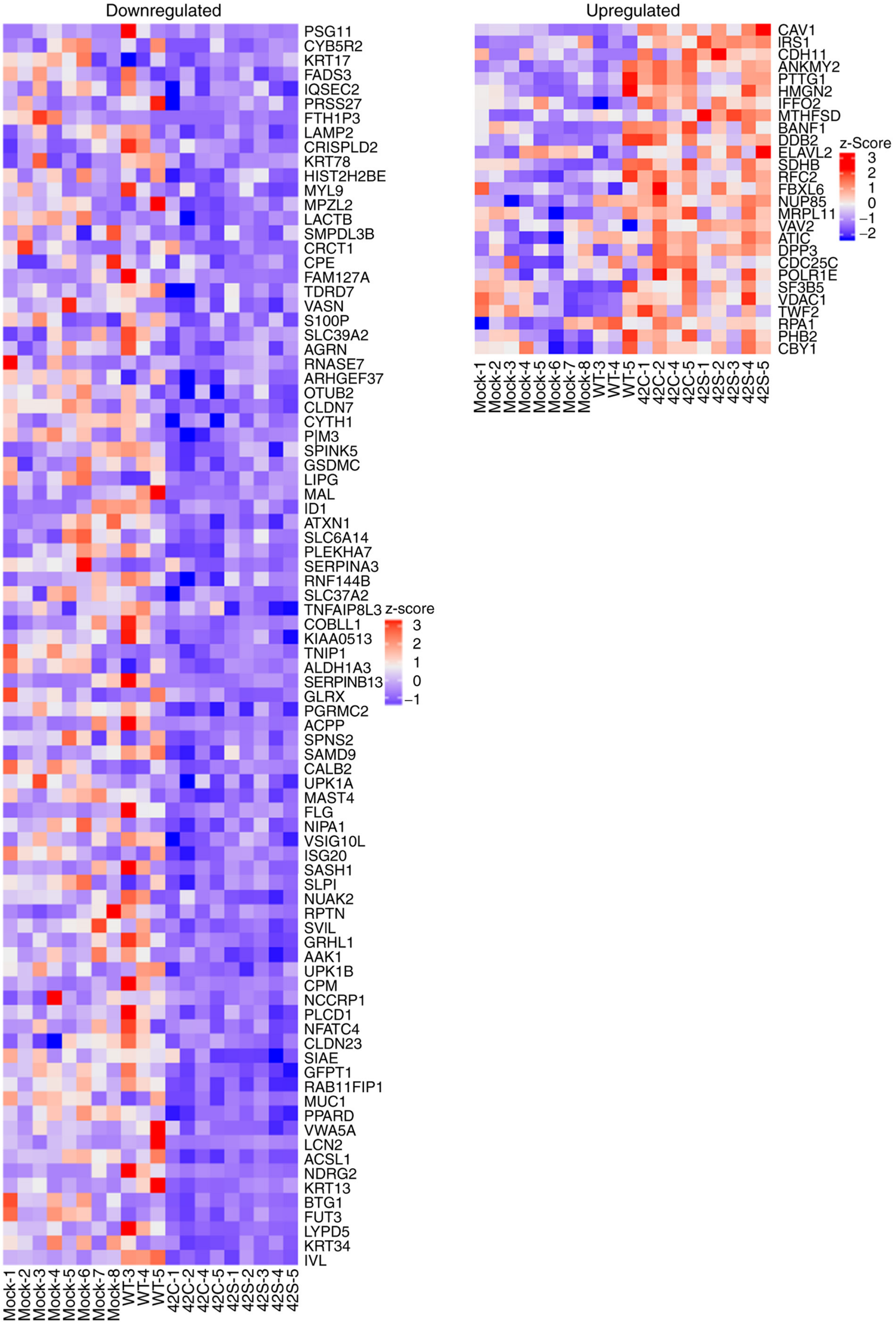

Table SI. The tumors subjected to histopathology and transcriptome sequencing.

Introduced vector	Sample ID	Pathology	RNAseq	Good reads	Uniquely mapped	Mapping on human	Mapping on mouse	Tumor rate (%)	Criteria Passed/NG
Mock	Mock-1	x	○	43,011,502	28,508,787	3,134,260	25,374,527	11.0	Passed
	Mock-2	x	○	43,999,830	31,143,655	5,363,072	25,780,583	17.2	Passed
	Mock-3	○	○	38,741,335	26,611,010	1,410,391	25,200,619	5.3	Passed
	Mock-4	○	○	37,849,948	25,805,921	3,553,336	22,252,585	13.8	Passed
	Mock-5	○	○	42,713,195	29,903,365	8,399,729	21,503,636	28.1	Passed
	Mock-6	○	○	34,337,685	24,342,738	5,198,692	19,144,046	21.4	Passed
	Mock-7	x	○	38,776,520	27,519,748	21,766,319	5,753,429	79.1	Passed
	Mock-8	○	○	38,066,105	27,286,425	21,423,436	5,862,989	78.5	Passed
WT	WT-1	○	x	36,185,604	25,610,289	38,999	25,571,290	0.2	NG
	WT-2	○	x	31,302,357	22,875,485	1,731	22,873,754	0.0	NG
	WT-3	x	○	34,437,645	25,742,989	22,922,847	2,820,142	89.0	Passed
	WT-4	○	○	39,314,638	29,097,066	24,116,698	4,980,368	82.9	Passed
	WT-5	○	○	39,924,991	28,908,953	5,562,383	23,346,570	19.2	Passed
Y42C	42C-1	○	○	43,838,287	30,569,870	2,389,900	28,179,970	7.8	Passed
	42C-2	○	○	41,635,723	29,640,538	2,802,156	26,838,382	9.5	Passed
	42C-3	○	x	39,261,333	28,934,139	27,947,610	986,529	96.6	NG
	42C-4	○	○	36,310,887	26,376,653	14,875,311	11,501,342	56.4	Passed
	42C-5	○	○	38,904,699	28,633,340	4,316,539	24,316,801	15.1	Passed
Y42S	42S-1	○	○	36,162,514	27,125,316	25,659,264	1,466,052	94.6	Passed
	42S-2	○	○	42,985,399	31,026,475	10,277,421	20,749,054	33.1	Passed
	42S-3	○	○	47,020,636	34,180,119	17,196,435	16,983,684	50.3	Passed
	42S-4	○	○	43,348,517	31,226,955	9,002,373	22,224,582	28.8	Passed
	42S-5	○	○	50,951,030	35,974,254	10,896,740	25,077,514	30.3	Passed

WT, wild-type; ○, included in analysis; x, excluded from analysis.

Table SII. Downregulated and upregulated genes after ROCK inhibitor treatment.

SIIA, Downregulated genes

#	Gene name
1	*A2ML1*
2	*A4GALT*
3	*AAK1*
4	*ABCA12*
5	*ACAP2*
6	*ACOT11*
7	*ACPP*
8	*ACSL1*
9	*AGRN*
10	*AHR*
11	*AKTIP*
12	*ALDH1A3*
13	*ANKRD22*
14	*ANKRD22*
15	*ANKRD35*
16	*AQP9*
17	*ARHGEF37*
18	*ARL5A*
19	*ASPRV1*
20	*ASS1*
21	*ATMIN*
22	*ATP12A*
23	*ATP6V0A1*
24	*ATXN1*
25	*BPIL2*
26	*BSPRY*
27	*BTG1*
28	*BZW1*
29	*C10orf116*
30	*C10orf54*
31	*C15orf52*
32	*C5orf46*
33	*C6orf15*
34	*C9orf169*
35	*CALB2*
36	*CALCOCO2*
37	*CALML5*
38	*CARD18*
39	*CASP4*
40	*CAST*
41	*CCDC132*
42	*CCDC64B*
43	*CD24*
44	*CD82*
45	*CEACAM6*
46	*CGN*
47	*CHMP4C*
48	*CIB2*
49	*CLCN3*
50	*CLDN23*
51	*CLDN7*
52	*CLIC3*
53	*CLINT1*

Table SII. Continued.

SIIA, Downregulated genes

#	Gene name
54	*CLIP1*
55	*CLTB*
56	*CNFN*
57	*CNKSR3*
58	*COBLL1*
59	*COL11A2*
60	*CPE*
61	*CPM*
62	*CRB3*
63	*CRCT1*
64	*CRISPLD2*
65	*CRNN*
66	*CST6*
67	*CST6*
68	*CWH43*
69	*CYB5R2*
70	*CYB5R3*
71	*CYP4B1*
72	*CYP4B1*
73	*CYP4F22*
74	*CYP4F3*
75	*CYTH1*
76	*DAAM1*
77	*DBNDD1*
78	*DBNDD2*
79	*DHRS11*
80	*DHRS3*
81	*DHRS9*
82	*DIP2B*
83	*DLG1*
84	*DNAJA4*
85	*DSC2*
86	*ENDOD1*
87	*EPHX3*
88	*ERP27*
89	*FA2H*
90	*FADS3*
91	*FAM127A*
92	*FAM129B*
93	*FAM135A*
94	*FAM3D*
95	*FAM43A*
96	*FLG*
97	*FLG*
98	*FTH1P3*
99	*FUT2*
100	*FUT3*
101	*FYTTD1*
102	*GAS6*
103	*GAS6*
104	*GCNT3*
105	*GDPD3*
106	*GFPT1*
107	*GLRX*

Table SII. Continued.

SIIA, Downregulated genes

#	Gene name
108	*GLTP*
109	*GLTPD1*
110	*GPR1*
111	*GPR56*
112	*GRHL1*
113	*GRHL3*
114	*GSDMA*
115	*GSDMC*
116	*HAL*
117	*HECTD3*
118	*HIP1R*
119	*HIST1H2AC*
120	*HIST1H2AE*
121	*HIST1H2BC*
122	*HIST1H2BD*
123	*HIST1H2BG*
124	*HIST2H2AA3*
125	*HIST2H2BE*
126	*HOPX*
127	*HPGD*
128	*HSPB1*
129	*HSPB8*
130	*HSPC159*
131	*ID1*
132	*IDS*
133	*IDS*
134	*IER3*
135	*IER5*
136	*IGFL2*
137	*IL1F10*
138	*IL1F5*
139	*IQSEC2*
140	*ISG20*
141	*ITPRIP*
142	*IVL*
143	*KAT2B*
144	*KAZN*
145	*KCNK1*
146	*KCNK12*
147	*KCTD21*
148	*KIAA0513*
149	*KIAA1468*
150	*KIAA1737*
151	*KLK11*
152	*KLK12*
153	*KLK13*
154	*KLK5*
155	*KLK5*
156	*KLK5*
157	*KLK6*
158	*KLK7*
159	*KLK7*
160	*KLK8*
161	*KPRP*

Table SII. Continued.

SIIA, Downregulated genes

#	Gene name
162	*KRT1*
163	*KRT10*
164	*KRT13*
165	*KRT17*
166	*KRT23*
167	*KRT34*
168	*KRT77*
169	*KRT78*
170	*KRT80*
171	*KRT80*
172	*KRT81*
173	*KRTDAP*
174	*LACTB*
175	*LAD1*
176	*LAMP2*
177	*LASS3*
178	*LCE3D*
179	*LCE3E*
180	*LCE6A*
181	*LCE6A*
182	*LCN2*
183	*LGALS8*
184	*LIPG*
185	*LIPM*
186	*LOC100131138*
187	*LOC100131138*
188	*LOC388564*
189	*LOC441052*
190	*LOC730081*
191	*LOR*
192	*LPHN2*
193	*LPXN*
194	*LRRC37A*
195	*LRRC8B*
196	*LSR*
197	*LY6G6C*
198	*LYPD3*
199	*LYPD5*
200	*LYPD5*
201	*MAL*
202	*MALL*
203	*MAMDC2*
204	*MAN2B2*
205	*MANSC1*
206	*MANSC1*
207	*MAP2*
208	*MAP2*
209	*MARCHF3*
210	*MAST4*
211	*MBOAT2*
212	*ME1*
213	*METRNL*
214	*METRNL*
215	*MFSD1*

Table SII. Continued.

SIIA, Downregulated genes	
#	Gene name
216	*MFSD6*
217	*MFSD6*
218	*MIR614*
219	*MLPH*
220	*MPZL2*
221	*MSRB3*
222	*MUC1*
223	*MUCL1*
224	*MYL9*
225	*NCCRP1*
226	*NCCRP1*
227	*NCF2*
228	*NCK2*
229	*NCRNA00087*
230	*NDRG2*
231	*NEBL*
232	*NFATC4*
233	*NIPA1*
234	*NLRX1*
235	*NPW*
236	*NRBF2*
237	*NSF*
238	*NUAK2*
239	*OBFC1*
240	*OSTF1*
241	*OTUB2*
242	*PCSK6*
243	*PERP*
244	*PGRMC2*
245	*PI3*
246	*PIM3*
247	*PITX1*
248	*PLA2G4E*
249	*PLA2G7*
250	*PLCD1*
251	*PLD5*
252	*PLEKHA7*
253	*PLEKHM1*
254	*PLOD2*
255	*POF1B*
256	*PPARD*
257	*PPP2R2B*
258	*PPP3CA*
259	*PRB1*
260	*PRB2*
261	*PRB4*
262	*PRDM1*
263	*PRICKLE2*
264	*PRKAR1A*
265	*PROM2*
266	*PRRT3*
267	*PRSS2*
268	*PRSS27*
269	*PSG11*

Table SII. Continued.

SIIA, Downregulated genes	
#	Gene name
270	*PSORS1C2*
271	*PTTG1IP*
272	*PYCARD*
273	*RAB11FIP1*
274	*RAB11FIP1*
275	*RAB9A*
276	*RAC1*
277	*RAET1E*
278	*RAET1G*
279	*RAET1L*
280	*RAET1L*
281	*RAP1GAP*
282	*RBM47*
283	*RDH11*
284	*RHCG*
285	*RIMBP3C*
286	*RNASE7*
287	*RNF144B*
288	*RPL21*
289	*RPTN*
290	*S100A11*
291	*S100A4*
292	*S100A4*
293	*S100A7*
294	*S100P*
295	*SAMD9*
296	*SASH1*
297	*SBSN*
298	*SC4MOL*
299	*SCEL*
300	*SCEL*
301	*SCNN1A*
302	*SDCBP2*
303	*SDCBP2*
304	*SDCBP2*
305	*SDR42E1*
306	*SDR9C7*
307	*SEC14L2*
308	*SERPINA3*
309	*SERPINB13*
310	*SERPINB3*
311	*SERPINB6*
312	*SGPP2*
313	*SH2D4A*
314	*SH3BGRL2*
315	*SH3KBP1*
316	*SH3KBP1*
317	*SHROOM3*
318	*SIAE*
319	*SLC12A8*
320	*SLC25A43*
321	*SLC37A2*
322	*SLC39A2*
323	*SLC39A8*

Table SII. Continued.

SIIA, Downregulated genes

#	Gene name
324	*SLC44A1*
325	*SLC44A3*
326	*SLC5A1*
327	*SLC6A14*
328	*SLPI*
329	*SMPDL3B*
330	*SNX2*
331	*SNX24*
332	*SORT1*
333	*SOX21*
334	*SPINK5*
335	*SPINK6*
336	*SPINK7*
337	*SPNS2*
338	*SPRR1A*
339	*SPRR2C*
340	*SPRR2E*
341	*SPRR2E*
342	*SPRR3*
343	*SPRR4*
344	*SPTLC3*
345	*SPTLC3*
346	*STK40*
347	*STRN*
348	*STX19*
349	*SULT2B1*
350	*SULT2B1*
351	*SVIL*
352	*TDRD7*
353	*TIMP2*
354	*TINAGL1*
355	*TMEM125*
356	*TMEM45A*
357	*TMEM45A*
358	*TMEM86A*
359	*TMSL3*
360	*TNFAIP8L3*
361	*TNIP1*
362	*TP53I3*
363	*TPD52L1*
364	*TPD52L1*
365	*TRADD*
366	*TRIM2*
367	*UGT1A6*
368	*UGT1A6*
369	*UGT2B7*
370	*ULBP2*
371	*UPK1A*
372	*UPK1B*
373	*USP6NL*
374	*VASN*
375	*VPS37C*
376	*VSIG10L*
377	*VWA5A*

Table SII. Continued.

SIIA, Downregulated genes

#	Gene name
378	*WFDC5*
379	*WFDC5*
380	*WSB2*
381	*XKRX*
382	*ZMIZ1*

SIIB, Upregulated genes

#	Gene name
1	*ADCK5*
2	*AGAP6*
3	*AMDHD2*
4	*ANKMY2*
5	*ANP32B*
6	*ARHGEF12*
7	*ATIC*
8	*BANF1*
9	*BNC1*
10	*C10orf2*
11	*C11orf17*
12	*C15orf42*
13	*C17orf81*
14	*C1orf105*
15	*CAV1*
16	*CBY1*
17	*CDC25C*
18	*CDH11*
19	*CHEK2*
20	*CKAP5*
21	*CLK2*
22	*CLUAP1*
23	*CPVL*
24	*CPVL*
25	*CREB5*
26	*DDB2*
27	*DDX28*
28	*DHX35*
29	*DPP3*
30	*DUSP5*
31	*ELAVL2*
32	*FAM156B*
33	*FAM82B*
34	*FAM86C*
35	*FBXL6*
36	*FCER1A*
37	*GALNTL4*
38	*GLDC*
39	*HERC5*
40	*HMGN2*
41	*HMGN2*
42	*HMGN2P46*
43	*HSPC157*
44	*IFFO2*

Table SII. Coninued.

SIIB, Upregulated genes	
#	Gene name
45	*IFT27*
46	*IL33*
47	*IRS1*
48	*JAG2*
49	*KCND3*
50	*LOC100507358*
51	*LRIG2*
52	*LY6H*
53	*MAD2L2*
54	*MRPL11*
55	*MRPL2*
56	*MSH6*
57	*MTHFSD*
58	*NLN*
59	*NUP85*
60	*OGG1*
61	*OPN5*
62	*PAOX*
63	*PGBD5*
64	*PHB2*
65	*PISD*
66	*PLCD4*
67	*POLR1E*
68	*PSMB8*
69	*PSME2*
70	*PTTG1*
71	*RFC2*
72	*RNPS1*
73	*RPA1*
74	*SDHA*
75	*SDHB*
76	*SEC22C*
77	*SEPHS2*
78	*SF3B5*
79	*SHMT1*
80	*SNORA24*
81	*SNORA61*
82	*SNORD14A*
83	*SNORD17*
84	*SNRPA*
85	*SSRP1*
86	*STAG3L3*
87	*THOC6*
88	*TMEM143*
89	*TSPAN4*
90	*TWF2*
91	*UTY*
92	*VAV2*
93	*VDAC1*
94	*WDR66*
